# A Review of the Institute of Medicine’s Analysis of using Chimpanzees in Biomedical Research

**DOI:** 10.1007/s11948-013-9442-7

**Published:** 2013-04-25

**Authors:** Robert C. Jones, Ray Greek

**Affiliations:** 1 Department of Philosophy, California State University, Chico, Chico, CA 95929-0730 USA; 2 Americans For Medical Advancement, 2251 Refugio Rd, Goleta, CA 93117 USA

**Keywords:** Animal experimentation, Ethics, Chimpanzees, Biomedical research, Biological complexity, Evolution

## Abstract

We argue that the recommendations made by the Institute of Medicine’s 2011 report, *Chimpanzees in Biomedical and Behavioral Research: Assessing the Necessity*, are methodologically and ethically confused. We argue that a proper understanding of evolution and complexity theory in terms of the science and ethics of using chimpanzees in biomedical research would have had led the committee to recommend not merely limiting but eliminating the use of chimpanzees in biomedical research. Specifically, we argue that a proper understanding of the difference between the gross level of examination of species and examinations on finer levels can shed light on important methodological and ethical inconsistencies leading to ignorance of potentially unethical practices and policies regarding the use of animals in scientific research.

## Introduction

In April 2011, the U.S. National Institutes of Health (NIH) asked the U.S. Institute of Medicine (IOM the health-arm of the privately funded National Academies) to form a committee to analyze the use of chimpanzees in biomedical and behavioral research. In December of that year, the committee published their findings in report, *Chimpanzees in Biomedical and Behavioral Research: Assessing the Necessity* (Institute of Medicine 2011) in which they recommend severely limiting the use of chimpanzees in biomedical research, and then, only if specific criteria are met. This article is an analysis of that report.

Though we applaud the fact that the IOM advise severely restricting the use of chimpanzees in such research, we believe that the report is methodologically and ethically confused. We argue that a proper understanding of evolution in terms of the science and ethics of using chimpanzees in biomedical research would have had led the committee to recommend not merely limiting but *eliminating* the use of chimpanzees in biomedical research. Specifically, we argue that a proper understanding of the difference between the gross level of examination of species and examinations on finer levels can shed light on important methodological and ethical inconsistencies leading to ignorance of potentially unethical practices and policies regarding the use of animals in scientific research.

## Background on and Specifics of the IOM Report

The NIH’s instructions to the IOM were clear and explicit. Specifically, the NIH requested the IOM to:

review the current use of chimpanzees for biomedical and behavioral research and: Explore contemporary and anticipated biomedical research questions to determine if chimpanzees are or will be necessary for research discoveries and to determine the *safety and efficacy* of new prevention or treatment strategies… to determine if chimpanzees are *necessary* for progress in understanding social, neurological, and behavioral factors that influence the *development, prevention, or treatment of disease* (Institute of Medicine 2011, 1 Emphasis added).

Notably, the NIH specifically requested an evaluation of the use of chimpanzees in predictive research, *not* basic research, an important distinction. *Predictive research* seeks to predict human response, while *basic research* seeks new knowledge regarding the material universe irrespective of whether that knowledge ever leads to practical advances (Greek and Greek 2010).For example, the determination of drug safety, toxicity, or efficacy, as well as the pathophysiology and natural history of disease, are a few of the ways that animals are used to model humans in*predictive* research, whereas the use of animals such as fruit flies to search for the functions of genes in the fruit flies is referred to as *basic* research. Usually when the topic of the ethics and efficacy of the use of animals in biomedical research is discussed, the focus is often on predictive research which claims explicitly that animal models can predict human response to drugs and disease (Shanks and Greek 2009; Shanks et al. 2009).The request from the NIH is typical in that the content revolves around the use of chimpanzees for *predictive* purposes, focusing specifically on the necessity and efficacy as predictive models of human disease.

Importantly, when used in the context of the use of animals in biomedical research to ascertain pathophysiological mechanisms or to develop better and safer drugs, the term *predict* does *not* mean merely a guess or a correlation. Nor does it refer to predictions made in order to test hypotheses. Very specific formulas are used in order to determine the *predictive value* of a modality in medical science and very specific terms are used to describe these parameters (see Table 1). Medical science requires positive predictive values and negative predictive values to be about 0.9 (90 %), otherwise the modality is not considered predictive. If a modality (such as an animal model) merely *correlates* with human data—even, say, 70 % of the time (most are far less, in the 5–50 % range)—this is considered not useful and does not qualify as predictive.

**Table 1:** Binary classification and means of calculating predictive values

	Gold Standard	
	GS+	GS−
Test		
T+	TP	FP
T−	FN	TN
Sensitivity = TP/(TP + FN)		
Specificity = TN/(FP + TN)		
Positive Predictive Value = TP/(TP + FP)		
Negative Predictive Value = TN/(FN + TN)		

*T*− test negative, *T*+ test positive, *FP* false positive, *TP* true positive, *FN* false negative, *TN* true negative, *GS*− gold standard negative, *GS*+ gold standard positive

## Conclusions and Recommendations of the Committee

The committee concludes that “[w]hile the chimpanzee has been a valuable animal model in past research, most current use of chimpanzees for biomedical research is unnecessary, based on the criteria established by the committee.” (Institute of Medicine 2011, 4) Further, the committee makes the following two recommendations:

- Recommendation 1: The NIH should limit the use of chimpanzees in biomedical research to those studies that meet the following three criteria:
  1. There is no other suitable model available, such as in vitro, nonhuman in vivo, or other models, for the research in question;
  2. The research in question cannot be performed ethically on human subjects; and
  3. Forgoing the use of chimpanzees for the research in question will significantly slow or prevent important advancements to prevent, control, and/or treat life-threatening or debilitating conditions.
  - Recommendation 2: The NIH should limit the use of chimpanzees in comparative genomics and behavioral research to those studies that meet the following two criteria:
    1. Studies provide otherwise unattainable insight into comparative genomics, normal and abnormal behavior, mental health, emotion, or cognition; and
    2. All experiments are performed on acquiescent animals, using techniques that are minimally invasive, and in a manner that minimizes pain and distress. (Institute of Medicine 2011, 6).

Though the IOM was directed to avoid ethical and financial considerations in the report, in reality the report is loaded both with ethical language and moral implications. For example, the report states:

The committee’s view is that the chimpanzee’s genetic proximity to humans and the resulting biological and behavioral characteristics not only make it a uniquely valuable species for certain types of research, but also demand a greater justification for their use in research than is the case with other animals. Reports over many decades have established the principles and guidelines dictating that animal subjects must be used in studies only where the risk to the health and welfare of humans is too great. Chimpanzees share biological, physiological, behavioral, and social characteristics with humans, and these commonalities may make chimpanzees a unique model for use in research. However, this relatedness—the closeness of chimpanzees to humans biologically and physiologically—is also the source of ethical concerns that are not as prominent when considering the use of other species in research. This is consistent with the 2010 European Union Directive, which notes that ethical issues are raised by the genetic proximity to human beings… ethics was at the core of any discussion […] on the continued used of chimpanzees in research. (Institute of Medicine 2011, 14)

In the next section, we closely examine this concept of “genetic proximity to humans,” a crucial justification of the IOM’s recommendation. We approach this from an evolutionary biology perspective and consider what this proximity implies as well as what it does not imply. We argue that this proximity means different things depending the level of examination. We continue this theme in light of the fact that animals are examples of complex systems and as such are subject to specific characteristics that influence what can be expected in terms of inter-species extrapolation.

## Levels of Examination

At the level of the periodic table, all life is similar, indeed in some sense, identical. That all humans are composed of carbon, hydrogen, oxygen, etc., is informative and can be quite useful in various domains of inquiry. However, it should be obvious that at this level of examination, using elemental similarity to determine how a patient will react to a drug or whether a particular animal species is sentientis not very useful. Similarly, examining life at the phylogenic level (e.g., noting that members of the Animalia and Plantae kingdoms are similar in that they are both all *alive*) takes place at a level of examination that is not helpful for predicting things like response to drugs and disease. For example, all members of the phylum Chordata have a heart that pumps blood to the tissues. As heart disease is one of the leading causes of morbidity and mortality among humans, one might expect a study of any given mammal to inform scientists about heart disease. However, such is not the case.(van der Worp and Macleod 2011; Fedorov et al. 2011; Greener et al. 2011; Gross 1985; Duff and McMillan 1949; Howard et al. 1972; Peters and Van_Slyke 1948 pp. 484, 500–501, 534–536; Shannon 1959 p 609]. Likewise, cancer affects humans and animals but the mechanisms and pathophysiology vary greatly. (Johnson et al. 2001; Leaf 2004; Brennan et al. 2010; Zielinska 2010; Editorial 2011; Brower 2011; Brody 2011; Rangarajan and Weinberg 2003).

An important and useful distinction can be made at this point regarding levels of examination. Levels of examination can be classified as either *gross* or *fine*.^1^ On the *gross* level, mammals have hearts that pump blood. However, as our analysis of hearts moves to a *fine* level, we find salient differences among mammals, specifically between humans and other mammals. For example, humans are unique in that we respond to certain genetic and environmental factors by depositing plaque in our coronary arteries. If a scientist sought to learn about coronary artery disease in humans by studying mice, he would be disappointed as most species are not susceptible to coronary artery disease (see references just above). Likewise, at the gross level, the human immunodeficiency virus (HIV) can infect humans and chimpanzees. However, at finer levels of examination we notice that the results of infection with HIV are different: humans die from HIV, while chimpanzees essentially come down with a cold-like illness for a few days. There are myriad differences in immune response that account for this despite the fact that on the gross level both species have immune systems that have much in common (Gardner and Luciw 1989; Ferrari et al. 1993; Fultz 1993; Johnston 2000).

The brain is another excellent example of similarities on the gross level but where dramatic differences become apparent with a finer level of examination. Brains in mammals are composed of essentially the same kinds of cells and the gross structure is laid out similarly among species. In all mammals, there exists an area that controls motor activity, an area responsible for sensation, balance, hearing and so forth. But the response of the brain to drugs and disease differs significantly (Barnes and Hayes 2002; O’Collins et al. 2006; Schnabel 2008; Enna and Williams 2009; Geerts 2009; Regenberg et al. 2009; Mogil 2009; Unknown 2010; McArthur 2011).^2^

Genetic similarity is used to justify an expectation of similar responses to drugs and disease and therefore for using certain animals to test drugs and explore mechanisms of disease. For example, chimpanzees are nearly 99 % identical to humans in terms of nucleotide sequences. However, with regard to testing, this similarity is meaningless as nucleotide sequence is but one factor that determines what function the gene actually has and when and for how long it carries out this function. A gene must be placed in the context of other genes, gene networks, regulatory genes, modifier genes, and proteins before a full description can be established. This finer level of examination is necessary for discovering useful information regarding drug and disease response.^3^

Thus, at the fine level, making claims based on “genetic proximity” is quite often as meaningless as pointing to similarities in anatomy and physiology and assuming similarity in structure translates to similarity in origin. The importance of the similarity is dependent upon the question being asked and the level of examination involved. Thus, the level of examination (or organization) is important.

## Evolution and Advances in Science

Futuyma famously notes that “[e]volution…is the central unifying concept of Biology. By extension, it affects almost all other fields of knowledge and must be considered one of the most influential concepts in Western thought” (Futuyma 1998). While this is generally accepted among scientists, such was not always the case. Darwin and Wallace’s idea fought for acceptance for decades. The early physiologists in France (including Claude Bernard, the father of our current paradigm of the use of animal models in drug and disease research) were among those that thought natural selection and descent with modification was simply wrong (Elliot 1987; Bernard 1957 (1865); LaFollette and Shanks 1994). Their belief, that all component parts in mammals were identical once size considerations were taken into account (Bernard 1957 (1865)), persists to this day.

However, even when the underlying assumptions are incorrect, advances in science can take place. For example, early morphologists like Cuvier rejected evolution in thinking that each category of animals was completely separate from all the others. Nonetheless, these researchers made significant pioneering advancements in science and made discoveries regarding phylogeny that, eventually, were interpreted as supporting evolution (Mayr 2002, 25). Importantly however, there are several differences between the early study of morphology and current study of human disease and drug reaction.

First, very little was known about phylogeny during Cuvier’s time whereas today much of what can be learned about the gross similarities among animals and humans has been uncovered. Despite its somewhat tautological nature this is not an insignificant point. In any field involving comparative anatomy and physiology the gross commonalities eventually are exhausted and the research modality must shift if more is to be learned in the most efficient way possible.

A second difference between the morphologists who denied evolution yet advanced science and today’s research is the level of examination. We now understand that drugs and disease act at the level of the gene or cell, not the level of the gross organ (although the effects are seen there). Hence, very small differences at the level of the gene negate the gross similarities in terms of drug development and disease research. Early anatomists and physiologists studied at the gross level where much similarity exists among species. The level of examination is still important in assigning gross characteristics. For example, in the 17th and 18th centuries, some human groups were not considered fully sentient beings just as many today do not consider animals sentient. A superficial examination of the brain and behavior refutes this notion. Thus, both the gross and fine level of examination are important depending on what question is being asked.

## Evolution of Human and Nonhuman Hominids

The line that led to humans diverged from the line that led to old world monkeys 25 million years ago (Mya) (Goodman 1999); the lines that led to humans and gorillas split ~18 Mya; the last common ancestor of humans and orangutans was ~13 Mya; and the human split from the line that led to chimpanzees and bonobos, ~6 Mya (about the same amount of time separating deer from giraffes) (Goodman 1999). We will refer to humans and the great apes as *hominids* with the great apes being nonhuman hominids (NHHs) (Harrison 2010). Humans are evolutionarily more closely related to NHHs than mice are to rats. Nevertheless, there are myriad differences between humans and NHHs that have biomedical significance. (See Tables 2 and 3 (Varki et al. 2011).)

**Table 2:** Human-specific changes in sialic acid biology–related genes (Varki et al. 2011)

Gene	Human-specific changes	Possible consequences for humans
*CMAH*	Human-specific *Alu*-mediated deletion eliminates exon 6, resulting in frame-shift and truncated inactive enzyme	Loss of Neu5Gc and excess of Neu5Ac expression on cell surfaces Corresponding effects on pathogen recognition and invasion Metabolic incorporation of Neu5Gc from diet, despite anti-Neu5Gc antibodies
*SIGLEC1*	Increased endogenous Neu5Ac-rich ligands in humans; enhanced frequency and broader expression pattern in macrophages	Increased likelihood of masking by endogenous Neu5Ac-rich ligands? Altered phagocytosis of Neu5Ac-expressing pathogens? Increased uptake of hypersialylated viruses by macrophages?
*SIGLEC5*	Expression suppressed on T cells; likely restoration of essential arginine residue for sialic acid recognition	Hyperresponsive phenotype of human T cells Possible role in propensity for diseases associated with T cell activation Interactions with group B *Streptococcus* type Ia “protein?
*SIGLEC14*	Likely restoration of essential arginine residue for sialic acid recognition; fusion/deletion population polymorphism	Loss of leukocyte activatory potential in homozygous null individuals?
*SIGLEC6*	Placental trophoblast expression	Expression levels increase with progress of labor Expression is further upregulated in preeclampsia (a human-specific disease)
*SIGLEC7* and *SIGLEC9*	Amino acid changes in V-set domain; adjusting of Neu5Gc to Neu5Ac recognition	Enhanced susceptibility to Neu5Ac-expressing pathogens that dampen innate leukocyte responsiveness?
*SIGLEC11*	Human-specific gene conversion; new expression in brain microglia	Altered interactions of microglia with neural cells? Altered response of microglia to infections?
*SIGLEC12*	Human-specific mutation of “essential arginine residue” for sialic acid recognition	Unknown
*SIGLEC13*	Human-specific deletion	Unknown
*SIGLEC16*	Human-specific inactivating mutation; population polymorphism	Altered interactions of microglia with neural cells? Altered response of microglia to infections?
*ST6GAL1*	Increased expression of Siaα2–6Galβ1-4GlcNAcβ1 termini in various cell types	Protection from avian influenza viruses, which prefer α2–3 sialic acid linkages, and susceptibility to human influenza viruses, which prefer α2–6 sialic acid linkages

*Neu5Ac* *N*-acetylneuraminic acid, *Neu5Gc* *N*-glycolylneuraminic acid

**Table 3:** Apparent differences between humans and nonhuman hominids (NHHs) in the incidence and severity of biomedical conditions^a^ and the potential role of sialic acid biology changes (Varki et al. 2011)

Medical condition	Humans	NHHs	Potential roles of sialic acid biology changes
*Definite differences*			
Myocardial infarction	Common	Very rare	Low Siglecs: increased immune reactivity? Dietary Neu5Gc accumulation in endothelium and atheromas
Interstitial myocardial fibrosis	Rare	Common	Different patterns of cardiac sialylation?
*Plasmodium falciparum* malaria infection	Susceptible	Resistant	Neu5Ac is the preferred merozoite ligand
Sexually transmitted bacterial diseases	Common	Very rare	Bacterial Neu5Ac engages Siglecs?
HIV infection progressing to AIDS	Common	Very rare	Low Siglecs: increased immune reactivity?
Foamy virus (spumavirus) infection	Rare	Common	Did anti-Neu5Gc antibodies eliminate?
*Probable differences*			
Human influenza A susceptibility	Variable	Often mild	α2-6-linked Sias on upper airways Low Siglecs: increased immune reactivity?
Hepatitis B/C late complications	Variable	Often mild	Low Siglecs: increased reactivity?
Alzheimer’s disease pathology	Common	Rare	Siglec expression in microglia?
Epithelial cancers (carcinomas)	Common	Rare?	Neu5Gc in carcinomas
Neu5Ac-expressing bacterial pathogens	Common	Rare?	Excess endogenous Siglec-1 ligands? Bacterial Neu5Ac engages inhibitory Siglecs?
Preeclampsia	Common	Rare?	Siglec-6 expression in placenta
Preterm labor	Common	Rare?	–
*Possible differences*			
Rheumatoid arthritis	Common	Rare?	Low Siglecs: increased immune reactivity? Neu5Gc in joints?
Bronchial asthma	Common	Rare?	Low Siglec: increased immune reactivity?
Early fetal wastage	Common	Rare?	–
Hydatidiform molar pregnancy	Common	Rare?	–
Endometriosis	Common	Rare?	Neu5Gc in endometrium?
Female iron deficiency	Common	Rare?	–
Major psychiatric diseases	Common	Rare?	–

*Neu5Ac* N-acetylneuraminic acid, *Neu5Gc* N-glycolylneuraminic acid

^a^Excludes disease differences due to obvious anatomical differences

It is certainly true that from an evolutionary standpoint, we expect there to be fewer differences between humans and chimpanzees than between humans and mice or humans and yeast. Nevertheless, because humans and our closest phylogenetic relatives are complex adaptive systems, we should expect small differences to be of great biomedical significance. This problem is compounded the further away one moves in terms of a common evolutionary ancestor. If chimpanzees cannot predict human response to drugs and disease to the precision necessary in medical science, it is even less likely that mice, even genetically modified mice, will fulfill this role.

Some biomedical differences among species can be explained simply by differences in anatomy, regardless of evolutionary history. Examples of these differences between humans and NHHs would include sinusitis, infection of air sacs (which humans do not have) in NHHs, sleep apnea, musculoskeletal disorders of the back, a larger head and smaller pelvic outlet present difficulties in childbirth for humans, sudden infant death syndrome (SIDS), varicose veins, acne, hemorrhoids, slower wound healing in humans, and inguinal hernias. Differences that cannot be explained by anatomy include various disease of the heart, sexually transmitted diseases, various neurological diseases, and various infectious diseases. In particular chimpanzees infected with HBV or HCV rarely develop hepatocellular carcinoma or chronic hepatitis (Varki et al. 2011; Walker 1997; Gagneux and Muchmore 2004; Bettauer 2010). (See Tables 2 and 3.)

One reason humans differ from NHHs involves sialic acid. (See Tables 2 and 3) Sialic acid is a family of sugars that have a 9-carbon backbone. They are found at the end of glycan chains that are located on the surface of cells. There are fewer than 60 genes involved in sialic acid synthesis. Ten of these genes are hot spots in evolution demonstrating significant differences between humans and NHHs (Varki et al. 2011).

## Biological Complexity

The evolution of new species must be placed into the context of complexity science. One reason why small differences in evolutionary history can result in profound differences in outcomes to perturbations is because animals and humans are complex rather than simple systems. A simple system can usually be defined and studied using Newtonian physics and can be expected to demonstrate a linear, or 1:1, response to perturbations. Simple systems are usually merely a sum of their parts and hence are also amenable to study by reductionism and determinism. Complex systems have very different characteristics (Csete and Doyle 2002; Sole and Goodwin 2002; Kitano 2002a, 2002b; Kauffman 1993; Ottino 2004; Alm and Arkin 2003; Goodwin 2001; Van Regenmortel 2002b, 2002a, 2004b, 2004a).

Complex systems are quite dependent upon initial conditions, as are chaotic systems. This fact is often overlooked for complex systems because the initial conditions for a complex system (such as, for example, financial markets), have been superseded by the outcomes of perturbations over the days and decades it has been in existence. In order to evaluate how the New York Stock Exchange will react to news of higher unemployment, for example, one does not need to understand the initial conditions of the NYSE when it was founded. Further, if one wishes to predict whether the Japanese Stock Exchange will respond to the same perturbation in the same fashion as the NYSE, one does not need to understand the initial conditions upon which it was founded. At the level of organization that is under study in such situations, the very distant past of the market is unimportant. However, if one wishes to predict the outcome to a perturbation for one living complex system, say a human, by using a second living complex system, say a mouse, then the initial conditions in the form of genetic makeup—including all the interactions, modifier genes, environmental influences, gene networks, and so on—must be considered. One very small difference in genetic makeup between the mouse and human can result in opposite reactions to a perturbation. In addition, complex systems are best-described using partial differential equations and all the values for the equations are not known. Thus predicting human response to drugs and disease based on mouse studies is unlikely in principle.

Complex systems are more than a sum of their parts; therefore reductionism has limits for evaluating a complex system. Another reason complex systems need to be studied as a whole is that they demonstrate emergent phenomena. Emergence is the appearance of a new trait or characteristic in a system that could not have been forecast even with complete knowledge of the component parts of the system. The level of examination is also important. Complex systems exhibit a hierarchy of organization and perturbations can result in opposite outcomes at different levels. The presence of feedback loops, environmental factors, redundancy, and robustness can also result in very different outcomes between two otherwise very similar complex systems.

Therefore, different species should be expected to react differently to the same perturbation to the system and this is has been confirmed empirically (LaFollette and Shanks 1995; LaFollette and Shanks 1996; Shanks and Greek 2009; Shanks et al. 2009; Sharp and Langer 2011; Chapman 2011; Giri and Bader 2011; Collins 2011; Caponigro and Sellers 2011; Leaf 2004; Ellis and Fidler 2010; Gura 1997; Sarkar 2009; American Paraplegia Society 1988; Littman and Williams 2005; Wall and Shani 2008; Smith et al. 1965; Smith and Caldwell 1977; Fletcher 1978) (Suter 1990 p 73; Lumley 1990 pp. 49–56; Heywood 1990 pp. 57–67). Moreover, merely changing a gene, either in the form of adding a human gene to a mouse or knocking out a gene in a mouse, should not be expected to substantially increase the predictive value of mouse models. This has also been confirmed empirically (LeCouter et al. 1998; Zutphen 2000; Morange 2001; Pearson 2002; Nijhout 2003; Van Regenmortel 2004b; Darlison et al. 2005; Shapiro 2007; Kieburtz and Olanow 2007; Young 2008; Enna and Williams 2009; Geerts 2009).

## The IOM Recommendations Versus Science

Given this discussion and a better understanding that animals are complex systems with different evolutionary trajectories, we can now analyze the scientific soundness of the committee’s recommendations. The committee states:

The committee cannot predict or forecast future need of the chimpanzee animal model and encourages use of the criteria established in this report when assessing the potential necessity of chimpanzees for future research uses… Having reviewed comparative genomics research, the committee concludes the chimpanzee may be necessary for understanding human development, disease mechanisms, and susceptibility because of the genetic proximity of the chimpanzee to humans. (Institute of Medicine 2011, 66).

Given our discussion thus far, it should be clear that despite the IOM recommendation, even a species as closely related to humans as the chimpanzee will not be able to reliably predict human response to drugs and disease. Comparative genomics has confirmed the existence of very small differences between closely related species. Empirical evidence also suggests that predicting response to drugs and disease is nearly impossible and this empirical evidence is supported and explained by evolution theory and complexity theory. It should be evident that the IOM recommendation ignores the best science currently available in the form of evolutionary biology, evolutionary and developmental biology, complexity science, and empirical evidence.

In terms of science, what the committee could have addressed is the successful use of chimpanzees as heuristic models, as a source of tissues for use in humans, as bioreactors, as modes for other chimpanzees, for example to test vaccines designed for chimpanzees in the wild, and other scientifically viable uses of chimpanzees. However, the committee was specifically asked to consider the use of chimpanzees as *predictive models* and in this area chimpanzees as models have a history of failure. *As we hope to have made clear, this failure is explained by the theory of evolution and complexity theory.* The fact that the committee advocates such a conclusion, and similar conclusions, casts serious doubt on the scientific validity of the report.

The committee reports that the following positions would not be acceptable for justifying research using chimpanzees:

- “The chimpanzee is immunologically, physiologically, anatomically, and/or metabolically similar to human beings.” This statement is too broad.
- “Chimpanzees have previously been used in safety studies for this class of drug.” This statement is not specific as to the science driving the decision. (Institute of Medicine 2011, 29).

Yet, the committee subsequently makes numerous such statements:

It has been suggested that this approach provides two potential advantages over monoclonal antibody production in other species. First, because the antibody protein sequences between the chimpanzee and the human are so similar, further subcloning and humanization of the chimpanzee antibody sequences are not needed, and the resulting antibodies can be used directly in humans without further work. Second, because the immune responses of the chimpanzee and the human are so similar, it is likely that chimpanzees would mount immune responses that are similar to analogous immune challenges seen in humans. The chimpanzee/human chimeric monoclonal antibodies produced in these manners have proven to be effective in both in vitro and in vivo assays to neutralize infectious viruses or to block the action of bacterial toxins… (37).

The presence of similarly activated underlying brain structures would suggest that chimpanzees could be used to model human communication development… (62).

The similarity in the neuroanatomy between the human and the chimpanzee may make it a model for neuropsychiatric disorders, for example, expressing human risk genes via viral vectors or from optogenetic methods that exploit the chimpanzee functional neuroanatomy. (Institute of Medicine 2011, 65).

It is difficult to remain consistent in one’s philosophy of science when that philosophy is not founded upon strong *science*. Without a strong appreciation for and adherence to evolutionary biology, avoiding claims based on *similarity* proves impossible for the committee.

The committee appears confused once again when it states that “[c]ontinued advances over the past decade in imaging, genetics, in vitro, and in silico models, and sophisticated rodent disease models have provided scientists with more tools that could be used in place of the chimpanzee” (Institute of Medicine 2011 p 29). First, rodent models cannot predict human response to drugs and disease any better than chimpanzee models (Greek and Greek 2010; Greek et al. 2012; Shanks and Greek 2009). Both fail as predictive models for human response to drugs and disease for the reasons outlined above. The committee frequently makes statements claiming that other animals can be used as replacements for chimpanzees because they function as well as chimpanzees. This is dubious as no species predicts human response to drugs and disease in humans.

Even other humans fail in this regard hence the current emphasis on personalized medicine (Greek et al. 2012). The evidence for this is quite convincing. Men differ from women in their response to drugs and disease (Holden 2005; Kaiser 2005; Klein and Huber 2010; Simon 2005; Wald and Wu 2010; Willyard 2009) and variation is also seen among ethnic groups (Cheung et al. 1997; Couzin 2007; Gregor and Joffe 1978; Haiman et al. 2006; Kalow 1991; Kopp et al. 2011; Spielman et al. 2007; Stamer and Stuber 2007; Wilke and Dolan 2011). No animal model more closely resembles a human than one monozygotic twin resembles another, yet even monozygotic twins vary in their response to drugs (Bruder et al. 2008; Dempster et al. 2011; Fraga et al. 2005; Javierre et al. 2010; Wong et al. 2005). Human variation in response to drugs and disease should inform society and the committee regarding the use of animal models to evaluate perturbations to the human system at a fine level of examination. Moreover, since individual humans react so differently to drugs and manifest different aspects of disease, the future of drug development will resemble figure 3 (Jørgensen 2011). This will not be possible using animal models.

Further, in vitro and in silico also fail in most cases, explaining why the failure rate for drugs in development is so high. Contrary to the committee’s statements that drug development is dependent upon animal models such as chimpanzees and other NHPs, the pharmaceutical industry as well as scientists in fields related to drug development, have opined that the animal model in general is a failure. This is born out by empirical evidence and failure rates (LaFollette and Shanks 1995; LaFollette and Shanks 1996; Shanks and Greek 2009; Shanks et al. 2009; Sharp and Langer 2011; Chapman 2011; Giri and Bader 2011; Collins 2011; Caponigro and Sellers 2011; Leaf 2004; Ellis and Fidler 2010; Gura 1997; Sarkar 2009; American Paraplegia Society 1988; Littman and Williams 2005; Wall and Shani 2008; Smith et al. 1965; Smith and Caldwell 1977; Fletcher 1978) (Suter 1990 p 73; Lumley 1990 49–56; Heywood 1990 pp. 57–67). In 2006, then US Secretary of Health and Human Services, Mike Leavitt stated, “[c]urrently, nine out of ten experimental drugs fail in clinical studies because we cannot accurately predict how they will behave in people based on laboratory and animal studies” (FDA 2006). The current focus in drug development is on developing ethical human-based testing and implementing it early in the development process (Kola and Landis 2004; Horrobin 2003; Seligmann 2004/5; Cressey 2011).

## Ethical Aspects of the Report

As we have mentioned, although the intent of the committee was to assess only “the scientific necessity of the chimpanzee as a human model for biomedical and behavioral research” (Institute of Medicine 2011, 14), the report quickly acknowledges that “any assessment of the necessity for using chimpanzees as an animal model in research raises ethical issues, and any analysis must take these ethical issues into account” (Institute of Medicine 2011, 14). In fact, the very principles guiding the report itself are clearly ethical in nature:

1. The knowledge gained must be necessary to advance the public’s health;
2. There must be no other research model by which the knowledge could be obtained, and the research cannot be ethically performed on human subjects; and
3. The animals used in the proposed research must be maintained either in ethologically appropriate physical and social environments or in natural habitats. (Institute of Medicine 2011, 26–27).

Thus, though the report claims to be primarily about science, *its central focus is actually on the ethical aspects of the use of chimpanzees in biomedical research*.

Given our discussion thus far, we would like in this section to focus on just one aspect of the ethical component of the report, namely, what we see as an inconsistency in arguing (as does the report) *both* that

1. chimpanzees are sufficiently *similar* to humans such that their use as research subjects may be necessary, and
2. chimpanzees are sufficiently *dissimilar* from humans with regard to the lack of possession of morally relevant capacities such that their use as research subjects is ethically warranted.

### The Argument From Moral Inconsistency

One recommendation that the report comes to is that

1. the use of chimpanzees in biomedical research is ethical only if necessary.

Hidden beneath, implicit in, and central to (a) (and thus, to the entire report itself) is a kind of moral argument. Let’s call this argument the *Morally Relevant Difference Argument* (MRDA). It looks something like this:

1. Humans and chimpanzees differ in their physiological, anatomical, and cognitive properties.
2. Some of these properties have moral significance. That is, some of these properties are morally relevant properties.
3. Thus, humans and chimpanzees differ in their possession of morally relevant properties and capacities.
4. This physiological, anatomical, and cognitive dissimilarity entails a difference in moral value.
5. The physiological, anatomical, and cognitive properties possessed by humans are of greater moral value than those possessed by chimpanzees.
6. Therefore, humans are of greater moral value than chimpanzees.

What’s important to recognize for our purposes is that the MRDA relies on the existence of certain differences between the species that translate into differences in moral worth [reflected in premise (5)].

However, there lies behind the report another crucial assumption, one we have already discussed, namely, that there exist evolutionary and physiological *similarities* between humans and chimpanzees, and that it is in virtue of these similarities that chimpanzees are valued instrumentally as (supposed) reliable predictive scientific models. Though the committee explicitly cautions against justifying the use of chimpanzees in biomedical research based on such similarities, as we have seen, the committee makes numerous statements regarding the scientific significance of such things as neuroanatomical (as well as antibody-protein-sequence) similarities between the species.

This kind of reasoning leads to what we will call the *Moral Inconsistency Argument* (MIA). The MIA looks something like this:

1. Humans and chimpanzees differ significantly in their physiological, anatomical, and cognitive properties such that humans have greater moral value than chimpanzees (per the MRDA).
2. Yet, humans and chimpanzees are quite significantly similar in their physiological, anatomical, and cognitive properties such that chimpanzees make valid and reliable predictive scientific models.
3. However, many of these same physiological, anatomical, and cognitive properties that are sufficiently *different* such that they bestow greater moral value on humans are the very same properties that, *within the same domain*, are sufficiently *similar* to warrant use of chimpanzees as valid predictive models of human pathology.
4. But to say that two species are both sufficiently similar and sufficiently dissimilar within the same domain based on the very same physiological, anatomical, and cognitive properties such that one species is given greater moral weight is morally inconsistent.
5. Lack of consistency is unacceptable.
6. Therefore, such justifications for the inferior moral status of chimpanzees and their use in biomedical research must be rejected.

We believe that the MIA rests on a confusion between levels of examination.

### How the MIA Rests on a Confusion

The MIA rests on the committee’s confusing the *gross* with the *fine* level of examination. Specifically, premise (1) of the MIA tells us that humans and chimpanzees *differ* significantly such that humans have greater moral value than chimpanzees, while premise (2) tells us that humans and chimpanzees are so significantly *similar* that chimpanzees make valid and reliable predictive scientific models.

The problem (and thus the source of the confusion in the MIA) is that the “difference” referred to in (1) occurs at the *fine* (i.e., the molecular/genetic) level, while the “similarity” discussed in (2) occurs at that *gross* level. Again, what we mean here by ‘gross’ level are traits such as the possession of organ and immune systems, and, most importantly with regard to the ethical question, *sentience*, i.e., the ability to experience pain and pleasure. Thus, as expressed by (4), the committee’s confusion with regard to the levels of examination with regard to the notion of similarity (as we have seen, a notion central to their recommendation) leads them to say that the two species (humans and chimpanzees) are both sufficiently similar and sufficiently dissimilar within the same domain based on the very same physiological, anatomical, and cognitive properties. Again, the claim that that the one species possesses greater moral status than the other based on such a confusion of levels of examination with regard to similarity is *morally inconsistent*.

While it is the case that some animals share with humans traits representative of a gross level of examination (e.g., sentience), at the fine level small differences can translate to opposite outcomes in terms of perturbations. Two otherwise seemingly identical complex systems manifest dramatically different response to disease and drugs secondary to very small differences apparent only upon finer levels of examination. That is, there exist at the fine level of examination important dissimilarities that, while not ethically important, are important in terms of the use of animal models to predict human response to drugs and disease.

To reiterate, it is correct to say, at the *gross* level, that humans, chimpanzees, and mice are sentient. However, as discussed, variation and differences in response to drugs and disease exists among ethnic groups, between men and women, and even between monozygotic twins. These kinds of variation are due to very small differences at the *fine* level of examination, and these differences can have major medical implications in terms of treatment and diagnosis. So, for example, though humans are sentient, the phenomenal aspects of pain may differ between men and women.

Again, the committee advocates the substituting of rats and mice in place of chimpanzees. However, at the gross level, humans, chimpanzees, and mice are similar in an important and morally relevant way, namely, that they are all sentient. The claim that mammalian vertebrates are sentient is uncontroversial and will not be argued for here (Bekoff 2007a, 2007c, 2007b, 2009, 2010; Edelman et al. 2005; Roughan and Flecknell 2001; Gentle 1992; Stevens 1992; Sneddon et al. 2003; LeDoux 2006) pp. 132–33). (For more see Jones 2013) Sentience, however, is *the* morally relevant property in consideration here. It is clear that the committee too quickly adopts an ethical position that ignores moral relevance of the possession of sentience and its expression across a wide range of species from humans to chimpanzees to mice. These conclusions are significant, particularly within the context of *moral individualism*.

## Moral Individualism

Rachels (1991) argues that when we take seriously a rejection of the view that humans are fundamentally and categorically different from other animals, we must also take seriously the implications of such a rejection as nothing less than a fundamental shift in the very project of ethics. Though acknowledging the fact/value gap,^4^ Rachels argues that even if there is no deduction of moral conclusions from factual premises, the fundamental assumptions of traditional morality are no longer tenable in light of evolutionary theory.

Specifically, since traditional morality is based on the assumption that species boundaries mark essential, hierarchical differences (with *Homo sapiens* at the top) and since evolutionary theory views species non-hierarchically with the notion of species itself being merely a pragmatic boundary, then the kind of categorical differences in moral worth and moral treatment foundational to traditional morality cannot be sustained. Whereas other philosophers writing on the moral status of animals argue that discrimination on the basis of species (i.e., *speciesism*) is morally arbitrary since species differences pick out no genuine morally relevant differences, Rachels goes further, claiming that if the species boundary marks no *real* boundary at all, then traditional morality’s correlation of such a boundary with moral value and significance is undermined from the start.^5^ If, as Mayr makes clear, thinking of organisms in typological terms is rendered obsolete by evolutionary theory, and since evolutionary biology gives primacy to the uniqueness of individuals within populations (Mayr 1994), then the idea that the moral status and treatment of individuals should be based on what is “normal” for their species should also be abandoned.

Rachels instead proposes an ethical framework he calls *moral individualism*, the view that “how an individual may be treated is determined, not by considering his group memberships, but by considering his own particular characteristics”^6^ (Rachels 1991, 173). Since humans and animals exhibit a complex pattern of similarities and differences, morality must respect this complexity. Morally relevant differences vary with the different kinds of treatment being considered. Thus, insofar as two individuals—regardless of their species—are similar, they should be treated similarly, while to the extent that they are different, they should be treated differently. On this view, “a difference between individuals that justifies *one* sort of difference in treatment might be completely irrelevant to justifying another difference in treatment” (Rachels and Rachels 2007, 24). So, for example, in some contexts, capacities such as intelligence and rationality are morally relevant properties (e.g., in deciding whether to admit someone to law school) and irrelevant in others (e.g., the treatment by a physician of a broken arm). This leads to moral individualism’s central principle of equality: “Individuals are to be treated in the same way, unless there is a difference between them that justifies a difference in treatment” (Rachels 1991, 96). With regard to animals specifically, the principle implies that “[o]ur treatment of humans and other animals should be sensitive to the pattern of similarities and differences that exist between them. When there is a difference that justifies treating them differently, we may; but when there is no such difference, we may not” (Rachels 1991, 197).

According to moral individualism then, it is not enough to simply *assume* (as does the committee) that there exist enough morally relevant differences between chimpanzees and mice such that mice can be used—where scientifically appropriate—as replacements for chimpanzees. For at the gross level, chimpanzees and mice are similar with regard to one morally relevant, salient property, namely, sentience, and it is this similarity in capacity that causes any such out-of-hand proclamations about the scientific suitability—and thus, the ethical significance—of the substitution of mice for chimps as ethically problematic.

## Conclusion

Recent advances in evolutionary biology, genetics, and evo devo explain why differences in the regulation and expression of genes, along with differences in alleles, convergent evolution, pleiotropy, modifier genes, alternative splicing, copy number variants, SNPs, and other mutations result in different outcomes among different species to the same perturbation. Moreover, as animals and humans are examples of complex adaptive systems the above genetic differences should be *expected* to result in different outcomes to drugs and disease. All of this plays out on the very fine level of examination of living systems and therefore, at this level of examination, the differences outweigh the similarities. The committee should have pointed this out and noted that empirically chimpanzees have failed to predict human response and therefore should no longer be used in such endeavors both. Such a recommendation would be consistent with human- and chimpanzee-based ethical concerns.

When studying the gross level however, where ethical concerns are relevant, the similarities have primacy. The denial of emotions in animals is actually a vestige of creationism. Evolution teaches a continuum. Creationism was and is all about distinct traits and interchangeable parts. Some component parts are shared, for example the presence of a heart and an immune system among mammals. Therefore, according to some creationists, the heart and immune system are fundamentally the same and hence interchangeable. Conversely, traits like sentience and the presence of a soul were thought to be present in humans only. This position was historically held by the animal-based research community as justification for using animal in research: animals are enough like us for experimentation purposes but lack sentience and souls therefore we have no moral obligation to them. This same reasoning was used to justify slavery, advocating the position that “lower races” suffered less than “higher ones” (Bending 2000, 123).

The committee ignores the fact that evolution is a continuum in terms of gross traits. It is highly unlikely that traits such as sentience arose *de novo* in hominids. The same is proving true for what have been thought uniquely human traits such as consciousness. As Ernst Mayr states:

How did human consciousness evolve? This is a question that psychologists love to ask. The answer is actually quite simple: from animal consciousness! There is no justification in the widespread assumption that consciousness is a unique human property. Students of animal behavior have brought together a great deal of evidence showing how widespread consciousness is among animals. (Mayr 2002, 282).

Evolution is a continuum. If morally relevant traits are a concern in terms of using chimpanzees in reserch then such should be the case for using mammals in general if not other classes and phyla of animals. By suggesting that one complex living system such as a chimpanzee, as well as other animals, can predict response to drugs and disease for another complex system, such as humans, the committee is retreading the old ground of “all organs are the same except for size.” By suggesting that chimpanzees can be replaced as predictive models by animals that are even more distantly related to humans the committee reveals not just a misunderstanding of evolution but of Complexity Theory and the current practice of science as regards drug and disease research. Accountability in medical research begins with sound science and a clear understanding of the ethical implications of one’s scientific assumptions and methodologies. We believe the IOM committee’s recommendations fail on both counts.

## Footnotes

1 Though an admittedly imprecise categorization, it will be adequate for our limited purposes.

2 For example, in 2002, Elan Pharmaceuticals and Wyeth-Ayerst were forced to halt Phase II studies on a vaccine for Alzheimer's disease identified as AN1792. The study was abandoned after discovering that 15 patients had developed severe inflammation of the brain. Some in the scientific community called for more testing on NHPs claiming that such research would have prevented AN1792 from going to human trials (Page 2002). In reality, AN1792 was tested on numerous species including NHPs and found to be safe (Marwick 2000) for those species.

3 AN1792 illustrated yet again that nucleotide similarity is not sufficient for predicting human response to drugs.

4 See Hume 1739/2007.

5 For a nice discussion of species anti-realism see (Mishler 2010).

6 For a solid, recent defense of moral individualism and the moral status of animals, see the work of Jeff McMahan, particularly (McMahan 2005).
